# Characterization of the Gut Microbiome Using 16S or Shotgun Metagenomics

**DOI:** 10.3389/fmicb.2016.00459

**Published:** 2016-04-20

**Authors:** Juan Jovel, Jordan Patterson, Weiwei Wang, Naomi Hotte, Sandra O'Keefe, Troy Mitchel, Troy Perry, Dina Kao, Andrew L. Mason, Karen L. Madsen, Gane K.-S. Wong

**Affiliations:** ^1^Department of Medicine, University of AlbertaEdmonton, AB, Canada; ^2^Department of Biological Sciences, University of AlbertaEdmonton, AB, Canada; ^3^BGI-ShenzhenShenzhen, China

**Keywords:** gut microbiome, 16S rRNA gene sequencing, shotgun metagenomics, bioinformatics, taxonomic classification, diversity analysis, functional profiling

## Abstract

The advent of next generation sequencing (NGS) has enabled investigations of the gut microbiome with unprecedented resolution and throughput. This has stimulated the development of sophisticated bioinformatics tools to analyze the massive amounts of data generated. Researchers therefore need a clear understanding of the key concepts required for the design, execution and interpretation of NGS experiments on microbiomes. We conducted a literature review and used our own data to determine which approaches work best. The two main approaches for analyzing the microbiome, 16S ribosomal RNA (rRNA) gene amplicons and shotgun metagenomics, are illustrated with analyses of libraries designed to highlight their strengths and weaknesses. Several methods for taxonomic classification of bacterial sequences are discussed. We present simulations to assess the number of sequences that are required to perform reliable appraisals of bacterial community structure. To the extent that fluctuations in the diversity of gut bacterial populations correlate with health and disease, we emphasize various techniques for the analysis of bacterial communities within samples (α-diversity) and between samples (β-diversity). Finally, we demonstrate techniques to infer the metabolic capabilities of a bacteria community from these 16S and shotgun data.

## Introduction

High-throughput comparative metagenomics enabled by development of next-generation sequencing (NGS) platforms (Mardis, 2008; Novais and Thorstenson, 2011) has led to an outburst of research endeavors that have rapidly advanced our understanding of the composition and function of bacterial populations in very diverse environments (Ley et al., 2006; Garrett et al., 2010; Caporaso et al., 2011; Bolhuis et al., 2014; Huttenhower et al., 2014a; Norman et al., 2014; Yoon et al., 2015). In the clinical context, the human gut microbiome has been the subject of intense investigation, which has revealed a sophisticated interplay between the microbiome and the host immune system and metabolism (Garrett et al., 2010; Brown et al., 2013; Huttenhower et al., 2014a; Martín et al., 2014; Broderick, 2015). For instance, it is well known that bacteria aid in many important metabolic pathways, including synthesis of essential compounds like secondary bile acids and short-chain fatty acids (Flint et al., 2012; Nicholson et al., 2012). Moreover, reduced diversity and/or imbalances in the gut microbiome have been associated with a variety of phenotypes, including obesity (Turnbaugh et al., 2009; Turnbaugh and Gordon, 2009), inflammatory bowel diseases (IBD) (Knights et al., 2013; Huttenhower et al., 2014b; Kostic et al., 2014; Norman et al., 2015), type II diabetes (T2D) (Qin et al., 2012; Hartstra et al., 2015), fatty liver disease (Arslan, 2014), and numerous additional disorders (Bhattacharjee and Lukiw, 2013; Dinan et al., 2014; Bajaj et al., 2015; Dash et al., 2015). The mechanisms whereby bacteria affect the host physiology are also well appreciated from a gene content/functional perspective. For example, both IBD and obesity are associated with enrichment of enzymes in the nitrate reductase pathway, the metabolism of choline and p-cresol, as well as the phosphotransferase system, required for assimilation of dietary carbohydrates (Greenblum et al., 2012; Levy and Borenstein, 2014). Bacteria able to synthesize short chain fatty acids, including acetate, butyrate, and propionate, have been found to be critical for colonocyte homeostasis, and their imbalance has been documented in diseases such like IBD and T2D (Qin et al., 2012; Brestoff and Artis, 2013; Kostic et al., 2014; Vital et al., 2014). For the most part, microbiome studies have focussed primarily on the structure and function of bacterial communities, fungi and viruses have received less attention thus far, but are starting to gain momentum (Reyes et al., 2010; Norman et al., 2014, 2015; Wang et al., 2015). There is also renewed interest in better understanding gaseous products from the gut microbiome, including carbon dioxide, hydrogen, methane and hydrogen sulfide (Pimentel et al., 2013). Importantly, methanogenesis from Archaea, mainly *Metanobrevibacter smithii*, is an important source of energy. It therefore influences metabolism and is associated with obesity, diabetes mellitus and other metabolic disorders (Pimentel et al., 2013; Barlow et al., 2015).

Most of the studies to understand bacterial population dynamics have been conducted with metagenomic approaches that are simple and cost-effective, although metatranscriptomic, proteomic, and metabolomic approaches are becoming popular too (Franzosa et al., 2014, 2015; Morgan and Huttenhower, 2014; Heinken and Thiele, 2015; Schaubeck et al., 2015; Yen et al., 2015). Together, these studies promise to provide a high-resolution picture of bacteria-host interactions that may lead to disease (Franzosa et al., 2015). Whole-metagenome shotgun analyses are accomplished by unrestricted sequencing of the genome of all microorganisms present in a sample (hereafter referred to as shotgun libraries); alternatively, inferences can be made by sequencing PCR amplicons from the ribosomal 16S RNA gene (hereafter referred to as 16S libraries), whose domain is restricted to bacteria and archaea (Janda and Abbott, 2007). Data generated by each of these approaches requires sophisticated computational methods and extensive hardware resources for their analysis (Gevers et al., 2012). This poses a significant challenge for microbiologists and clinical researchers interested in diverse aspects of the microbiota. Fortunately, the open-source software community has been diligent in developing user-friendly bioinformatics tools required for the analyses of bacterial NGS datasets. This article provides a compendium of good practices for the analysis of NGS microbiome libraries sequenced with the MiSeq platform but, for the most part, our suggestions are applicable to data generated with other NGS platforms. Using gut microbiome datasets specially designed to illustrate the strengths and weaknesses of 16S or shotgun libraries, we describe several methods for performing taxonomical classification of bacterial sequences, assessment of bacterial diversity within and between samples, and inference of the metabolic capabilities associated with the bacterial microbiome.

## Pre-processing to eliminate uninformative data

Removal of adapters, PCR primers and low quality bases is essential for effective analyses of NGS libraries, and a variety of user-friendly tools have been developed for this purpose. The current Illumina platforms output quality scores “Q” that fit into a 0–41 scale (Q10 corresponds to 1 expected error for every 10 sequenced bases; Q20 = 1 error for every 100 bases, and so on). Setting a quality threshold remains at the researcher's discretion; however, it is good practice to use only those sequences with the highest possible quality. In our experience, sacrificing sequences with low quality scores often improves the accuracy of the analyses by a significant margin. The gain in precision by trimming data is more significant for 16S data than it is for shotgun data, as clustering algorithms have been designed to detect minor differences along the sequence of the 16S rRNA gene. Most sequencing platforms are capable of performing paired-end sequencing. This means that both ends (end1 and end2) of the library insert are sequenced separately. End1 and end2 may or may not overlap and together are referred to as a “read.” With Illumina chemistry, bases at the front (5′ end) of each sequence generally exhibit higher quality than those at the back (3′ end) (Supplemental Figure 1); however, in the case of 16S libraries, the primers used for amplification can also generate regions of low quality at the front of each sequence. For shotgun data it is recommended to use trimming software that remove low-quality bases from both termini of each sequence, like cutadapt (Martin), sickle (Joshi and Fass, 2011), or fastqMcf (Aronesty, 2011). For 16S rRNA gene sequences, it is advisable to trim sequences along the entire length, starting from the 5′ end and using a quality threshold as high as possible, while leaving sufficient sequences to perform the analyses. Assembly of overlapping paired end sequences is advisable as long as the quality of overlapping regions is high enough to generate a consensus sequence with high quality scores.

## Taxonomical classification of bacterial sequences

Precise taxonomy assignments based on sequence alignments remain a computational challenge for both 16S and shotgun libraries, because of the short NGS read lengths. Prior to taxonomic classification, gene marker amplicon sequences, like regions of the bacterial 16S rRNA gene, are clustered by two main approaches (Sun et al., 2012; Chen et al., 2013). First, sequences can be clustered into phylotypes according to their similarity to previously annotated sequences in a reference database (Liu et al., 2008). Second, operational taxonomic units (OTUs) can be constructed by clustering sequences *de novo*, purely based on their similarity (Schloss and Westcott, 2011; Sun et al., 2012), which is computationally much more intensive. A hybrid method that combines both approaches is therefore recommended. In all cases, an arbitrary similarity threshold is used to differentiate clusters. The 99% similarity threshold is generally accepted as a good proxy for species (Stackebrandt and Ebers, 2006). However, this threshold is often insufficient to discriminate between closely related species, such as different members of the Enterobacteriaceae, Clostridiaceae, and Peptostreptococcaceae families. Importantly, higher resolution analytical tools have been published that overcome some of the limitations associated with clustering algorithms (Eren et al., 2013, 2014; Tikhonov et al., 2015).

Comprehensive reference databases have been compiled for annotation of sequenced bacteria metagenomes. For 16S rRNA genes, this includes the Greengenes database (DeSantis et al., 2006), the Ribosomal Database Project (RDP) (Cole et al., 2014), and SILVA (Quast et al., 2013). In addition to their extensive catalogs of curated 16S rRNA sequences, available for downloading, each of those portals also offers a series of bioinformatics tools for analysis of NGS sequences. Comprehensive analysis servers like MG-RAST are also publicly available, which already contain updated databases for annotation purposes (Meyer et al., 2008). More specifically, the human microbiome project (HMP) keeps a curated collection of sequences of microorganisms associated with the human body, including eukaryotes, bacteria, archaea and viruses, from both shotgun and 16S sequencing projects (C. Human Microbiome Project, 2012a,b). One of the approaches to increasing the resolution of taxonomical classification of sequences is to compile databases containing only the sequences likely to exist in the environment under study. For example, specialized databases comprising only members of the human intestinal microbiota (Ritari et al., 2015; Forster et al., 2016) have been created.

Robust bioinformatics approaches have also been developed for analysis of shotgun data (Riesenfeld et al., 2004; Schloss and Handelsman, 2008; Wu and Eisen, 2008; Huson et al., 2011; Boisvert et al., 2012; Gevers et al., 2012; Kultima et al., 2012; Namiki et al., 2012; Segata et al., 2012). Unique clade-specific marker genes (Mende et al., 2013) and lowest common ancestor (LCA) positioning approaches are among the most popular. For the former, a gene marker catalog is pre-computed from previously sequenced bacterial genomes and sequences are taxonomically classified by querying the catalog. For the LCA approach, pre-aligned sequences are hierarchically classified on a taxonomy tree using a placement algorithm (Aho et al., 1973; Huson et al., 2011). Sequences that surpass a dissimilarity threshold (bit-score) are progressively placed on higher taxonomy levels.

## Validation of bioinformatics approaches in bacterial communities

To demonstrate some of the most common approaches and pipelines used for taxonomy assignments in 16S and shotgun libraries, we created an artificial bacterial population using DNA from *Salmonella enterica, Streptococcus pyogenes, Escherichia coli, Lactobacillus helveticus, Lactobacillus delbrueckii, Lactobacillus plantarum, Clostridium sordelli, Bacteroides thetaiotaomicron, Bacteroides vulgatus, Bifidobacterium breve*, and *Bifidobacterium animalis*. We then constructed 16S and shotgun libraries in parallel using the NEXTflex 16S V4 Amplicon-Seq (BioO Scientific) and the Nextera XT (Illumina) kits, respectively. The raw data of all libraries generated during this study is publicly available at the Sequence Read Archive (SRA) portal of NCBI under accession number SRP059928.

**Figure 1:**
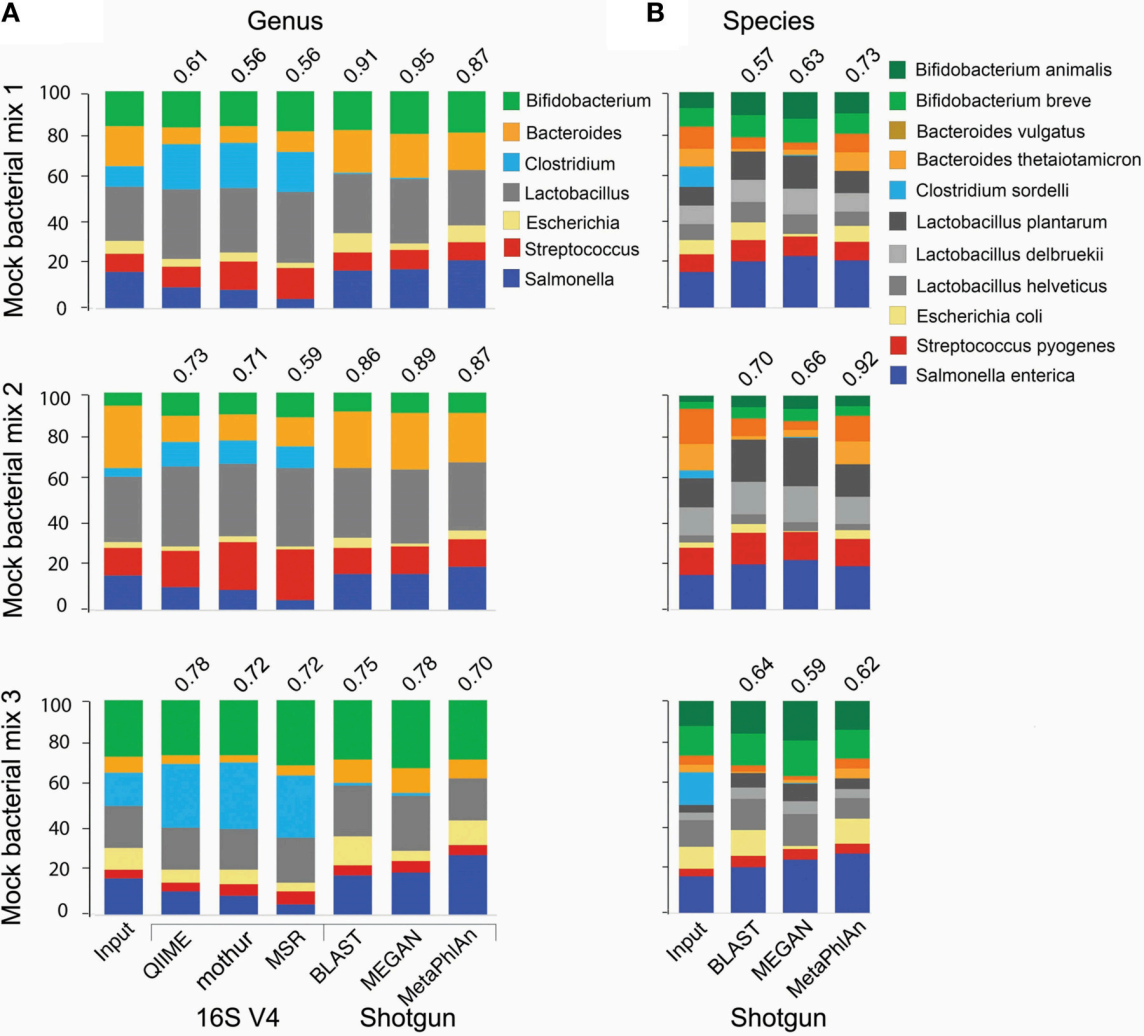
**Comparison of taxonomic analyses of a low complexity artificial microbial population using 16S amplicon or shotgun metagenomic approaches**. Eleven bacterial species (representing 7 genera) were cultured under standard laboratory conditions. DNA was extracted using the FastDNA spin kit for feces (MPBio). 16S amplicon and shotgun metagenomics libraries were constructed using the NEXTflex 16S V4 Amplicon-Seq (BioO Scientific) and the Nextera XT (Illumina) kits, respectively. Libraries were paired-end sequenced on a MiSeq sequencer using a 500-cycle kit. For 16S libraries, sequences were trimmed with the “split_fastq_libraries.py” script from QIIME. Default parameters were used, with the exception that the quality threshold for trimming was raised to 30. PCR primer sequences were trimmed with in-house Perl scripts. Shotgun metagenomics libraries were trimmed with the fastqMcf tool, and a quality threshold of 15. The relative abundance of each species was determined with the software indicated at the bottom of the bar graph, using default parameters, at the genus **(A)** or species **(B)** levels. The Pearson correlation coefficient between the expected (Input) relative abundance and the classification performed by each program is indicated on top of the bar graph.

For the analysis of 16S amplicon libraries, we evaluated QIIME (Caporaso et al., 2010; Navas-Molina et al., 2013) and mothur (Schloss et al., 2009), the most widely adopted pipelines, and the MiSeq Reporter v2.5 (MRS; the software developed by Illumina and accompanying the MiSeq instrument) pipeline, all with default parameters. At the genus level, all pipelines produced similar results, but the Pearson correlation coefficient between the expected (input) and obtained relative abundance was somewhat higher for QIIME (Figure 1A). We therefore selected QIIME for our subsequent analyses; however, we do not discourage the use of mothur, which is also a reliable pipeline. None of the 16S pipelines performed satisfactorily at the species level.

**Figure 2:**
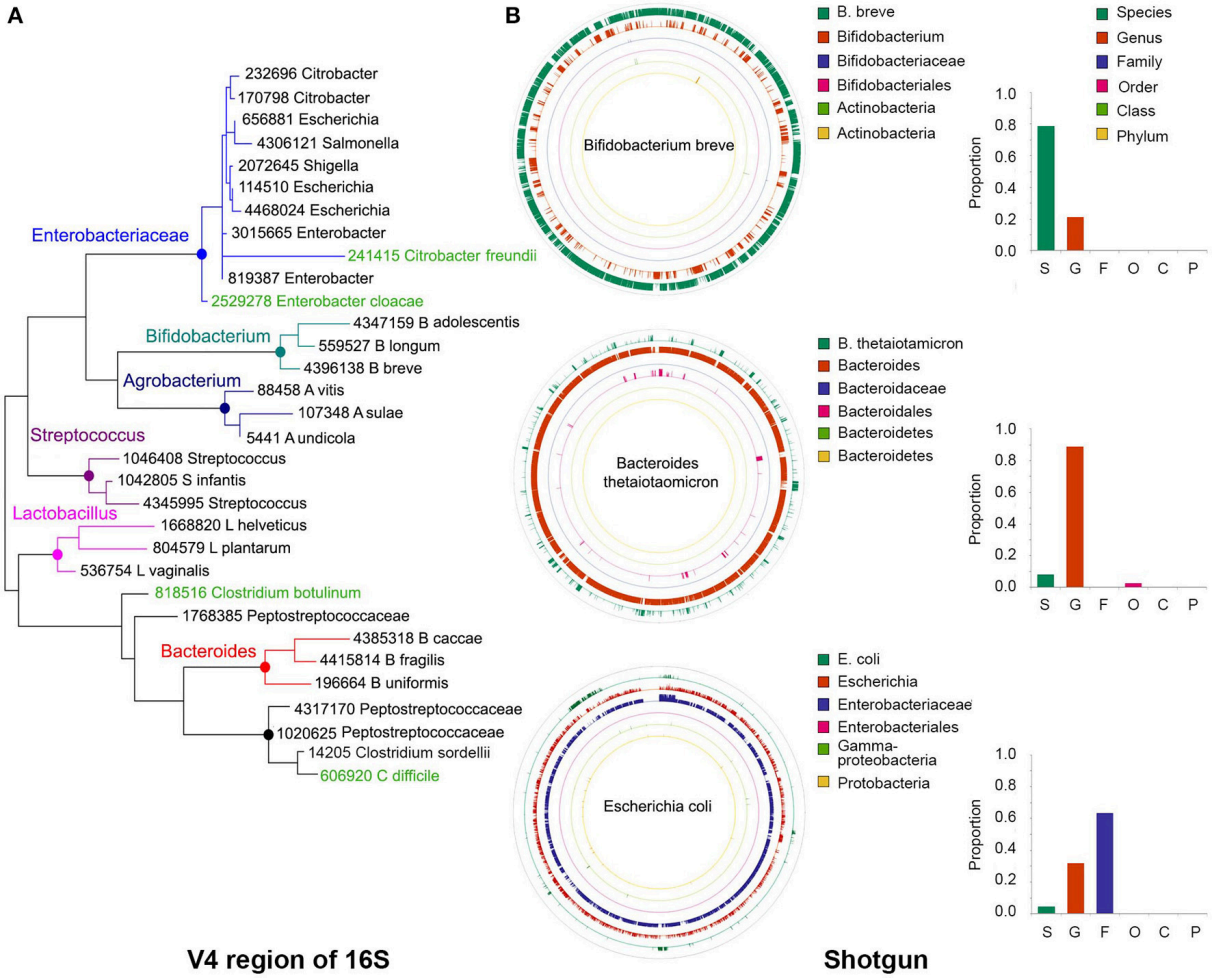
**Precision of taxonomy assignments is affected by highly similar sequences in different taxa. (A)** For the 16S libraries described in Figure 1, sequences were clustered into operational taxonomic units (OTUs) using a 97% similarity threshold and taxonomy assignments were performed with the RDP classifier. Sequences from OTUs classified as Bifidobacterium (*n* = 3), Agrobacterium (*n* = 3), Streptococcus (*n* = 3), Lactobacillus (*n* = 3), Bacteroides (*n* = 3), Peptostreptococcaceae (*n* = 4), or Enterobacteriaceae (*n* = 9) were randomly extracted and aligned to the Greengenes database to extract the closest relative (best hit). In addition, we included Greengenes 16S rRNA gene sequences (in green) from *Clostridium difficile* and *C. botulinum* as reference for Peptostreptococcaceae and *Citrobacter freundii* and *Enterobacter cloacae* as reference for Enterobacteriaceae. The V4 region of the 16S rRNA gene was cropped from the Greengenes sequences to construct a phylogenetic tree with MEGA-6, using UPGMA hierarchical clustering and 10,000 bootstraps. **(B)** Sequences from our bacterial populations in Figure 1 were aligned against the NCBI nt and human microbiome project (HMP) databases to identify the most similar reference genome. For each bacterium, a simulated library was created by segmenting the reference genome sequence into 500 nt stretches (250 nt paired ends in a head-to-tail orientation), iterating the process to generate ~1.5 million sequences. This simulated library was aligned back to the reference genome and the taxonomy resolved with MEGAN5. As examples, we show the reads classification of *Bifidobacterium breve, Bacteroides thetaiotamicron*, and *Escherichia coli*, which accumulated a large proportion of reads that could be resolved at the species, genus or family levels, respectively. Color-matched bars on the right show the proportion of reads accumulated at each level for these particular examples. S, species; G, genus; F, family; O, order; C, class; P, phylum.

We conducted taxonomy assignments using end1, end2, or both paired ends. When using Illumina chemistry, end1 typically exhibits higher quality than end2 (Supplemental Figure 1); accordingly, end1 provided a somewhat more accurate classification than end2 or paired ends (Supplemental Figure 2). However, the V4 variable region of the 16S rRNA gene is relatively short and in most cases will be covered by any one of the two ends (250 nt in this case); as such, these results may only reflect the higher quality of end1. For single ends, the best results were obtained with the *pick_open_otus.py* script from QIIME to cluster sequences (Supplemental Figure 2). Chimeric sequences can be artifactually generated when PCR amplification of the 16S region of interest is incomplete and the resultant partial sequences serve as primers that recombine with heterologous molecules containing a similar 3′ moiety. Several bioinformatics approaches have been developed for detection and removal of chimeric sequences. We used the USEARCH tool (Edgar, 2010) to remove chimeras. However, a desirable approach is to prevent formation of such chimeras *in vitro*, using high fidelity amplification protocols like LEA-Seq (Faith et al., 2013). Sequences were initially clustered into phylotypes using the Greengenes database of 16S rRNA sequences (DeSantis et al., 2006) as reference, while more dissimilar sequences were clustered *de novo* into OTUs. Taxonomy was then assigned using the RDP classifier, using the UCLUST method (Wang et al., 2007). RTAX (Soergel et al., 2012), a method embedded in QIIME, and UPARSE (Edgar, 2013) are algorithms especially designed to take advantage of mate pairs information. For paired-end analysis, the UPARSE pipeline (Edgar, 2013) produced more satisfactory results than the RTAX method (Soergel et al., 2012; Supplemental Figure 2). Irrespective of the method used for clustering, we found a consistent over-representation of sequences in the *Clostridium* and *Lactobacillus* genera. These two genera contain sequences that are perfectly complementary to the primers used for amplification, while at least one mismatch is found in the rest of genera included in our experimental (mock) bacterial population. This demonstrates how subtle differences in primer binding sites within the 16S rRNA gene sequences lead to biased estimates of relative abundance. Other primers have been reported to present biases, for instance the primer pair 27F/338R results in underrepresentation of *Bifidobacterium* (Martínez et al., 2009; Kuczynski et al., 2010). In our study, the detection of some *Clostridium, Escherichia* and *Salmonella* sequences was only possible after computational extraction of representative sequences of OTUs and blasting them against both the nr/nt and the 16S ribosomal RNA databases from NCBI. In general, sequences in the *Enterobacteriaceae* family and the *Clostridiales* order were poorly resolved using the 16S V4 or V3-V4 regions (Figure 2A), and this seems to be the case with *Enterobacteriaceae* for other 16S variable regions as well (Chakravorty et al., 2007).

For shotgun libraries, we compared BLAST top hits, the MEtaGenome ANalyzer MEGAN5, and Metagenomic Phylogenetic Analysis (MetaPhlAn) approaches; however, we do acknowledge that many other excellent tools have also been developed, including PhymmBL (Brady and Salzberg, 2009, 2011), PhyloSift (Darling et al., 2014), MOCAT (Kultima et al., 2012), Kraken (Wood and Salzberg, 2014), CLARK (Ounit et al., 2015), and kallisto (Schaeffer et al., 2015). BLAST top hits corresponded to the correct genus in all instances (Figure 1A), but there were inaccuracies at finer resolutions. For example, some *C. sordelli* sequences were erroneously assigned to *C. difficile* or *C. botulinum* because no reference genome was available at the time we conducted the alignments. MEGAN5 (Huson et al., 2011) hierarchically classifies pre-aligned sequences on a taxonomy tree using an LCA algorithm. As BLAST can be prohibitively slow, the LAST aligner was used in comparison for the same analysis (Kielbasa et al., 2011). LAST alignments were several orders of magnitude faster than BLAST, with comparable sensitivity (Supplemental Figure 3). The LAST-aligned sequences were fed to MEGAN5 for taxonomic assignments (Huson et al., 2011). Classification with LAST/MEGAN5 was as accurate as BLAST top hits at the genus level (Figure 1A). Lastly, we used MetaPhlAn, which infers taxonomy based on unique clade-specific marker genes. MetaPhlAn classification at the genus level was as accurate as the one performed by the other two tools (Figure 1A). The three tools correctly classified all species included in our mock populations and also provided a good approximation to their expected relative abundance (Figure 1B), but MetaPhlAn outperformed the other two tools in terms of precision and speed. Furthermore, utilization and installation of MetaPhlAn is much simpler than BLAST or MEGAN5 and it requires less computational processing.

## Sequences with low resolution cannot be classified at the species level

Resolving the taxonomy of 16S rRNA gene sequences can be problematic based on a limited segment of the 16S rRNA gene, such as the V4 region. In many cases, the sequence to be classified is nearly identical to several other bacterial sequences in the reference database. Similarly, for shotgun metagenomic analyses, when only parts of the bacterial genome are recovered, the classification at a taxonomic level will depend on the degree of conservation of the available sequences. Thus, the taxonomy of species that contain highly similar sequences will be more difficult to resolve, and the analyses will accrue a larger proportion of reads at the higher levels of the taxonomy tree.

For instance, the phylogenetic tree depicted in Figure 2A was built using the V4 region of few representative sequences in the Greengenes database (DeSantis et al., 2006; see Figure 2 caption for details). It can be seen that sequences in some genera form discrete branches on the tree, such as *Lactobacillus, Streptococcus, Bifidobacterium*, and *Bacteroides*. Other more closely related bacteria intertwine and cannot be delineated solely on the basis of their differences along the V4 region, such as those within the *Enterobacteriaceae* and *Peptostreptococcaceae* family. It has been reported that for ~42% of bacterial genera there will be pairs of sequences within genus that cannot be easily separated because their 16S rRNA gene sequences are more than 97% similar (Vetrovsky and Baldrian, 2013).

In a taxonomy tree, the lowest common ancestor of two taxa, *a* and *b*, is the immediate upper node that includes *a* and *b* as descendants. When a sequence aligns equally well to nodes *a* and *b*, that sequence will be annotated with the taxonomy corresponding to the lowest common ancestor, which is less accurate but more certain. Using the LCA approach, the lack of resolution of bacterial sequences in certain parts of the genome will also affect the taxonomic classification of shotgun libraries. For example, in bacteria with highly divergent genomes like *Bifidobacterium breve*, a large proportion of the genome can be resolved at the species level (Figure 2B, green outer circle), whereas in other genomes like those of *Bacteroides thetaiotamicron* and *Escherichia coli*, the majority of their sequences can only be resolved at the genus (Figure 2B, orange ring) and family (Figure 2B, purple ring) levels, respectively. MetaPhlAn does not suffer from this problem, as marker genes are chosen based on their uniqueness, with the caveat that sufficient sequences are needed to warrant their representation in shotgun libraries.

In general, classification of whole metagenome sequences improves when more dissimilar regions of the genomes, with greater discriminatory power, are included in the sequenced pool. When relatively large amounts of sequences are available, it is convenient to assemble individual reads into larger fragments, technically known as contigs, which are more amenable for taxonomic classifications. A series of software to assemble metagenomics data have been developed including Ray Meta (Boisvert et al., 2012), MetaVelvet (Namiki et al., 2012), MetaQUAST (Mikheenko et al., 2015), and MetAMOS (Treangen et al., 2013), among others. To increase efficiency, it is also possible to combine different samples in a single assembly procedure, while maintaining the ability to trace the origin of each assembled read.

## Assessment of required sequencing depth

For illustration purposes, we prepared a series of samples of progressively greater complexity. At the low-end, we sequenced the metagenome associated with grains of Kefir, a form of fermented milk with probiotic properties (Nielsen et al., 2014; Supplemental Figure 4). The higher complexity libraries included stool samples from subjects affected by Crohn's disease, *C. difficile* infection, and a healthy individual. For comparison, we cultured three experimental (mock) bacteria communities containing 19 species from 12 genera (Mix7-9). All libraries were sequenced at an average depth of ~ 8.5 × 10^5^ paired-end reads (minimum 1.57 × 10^5^; maximum 1.67 × 10^6^).

**Figure 3:**
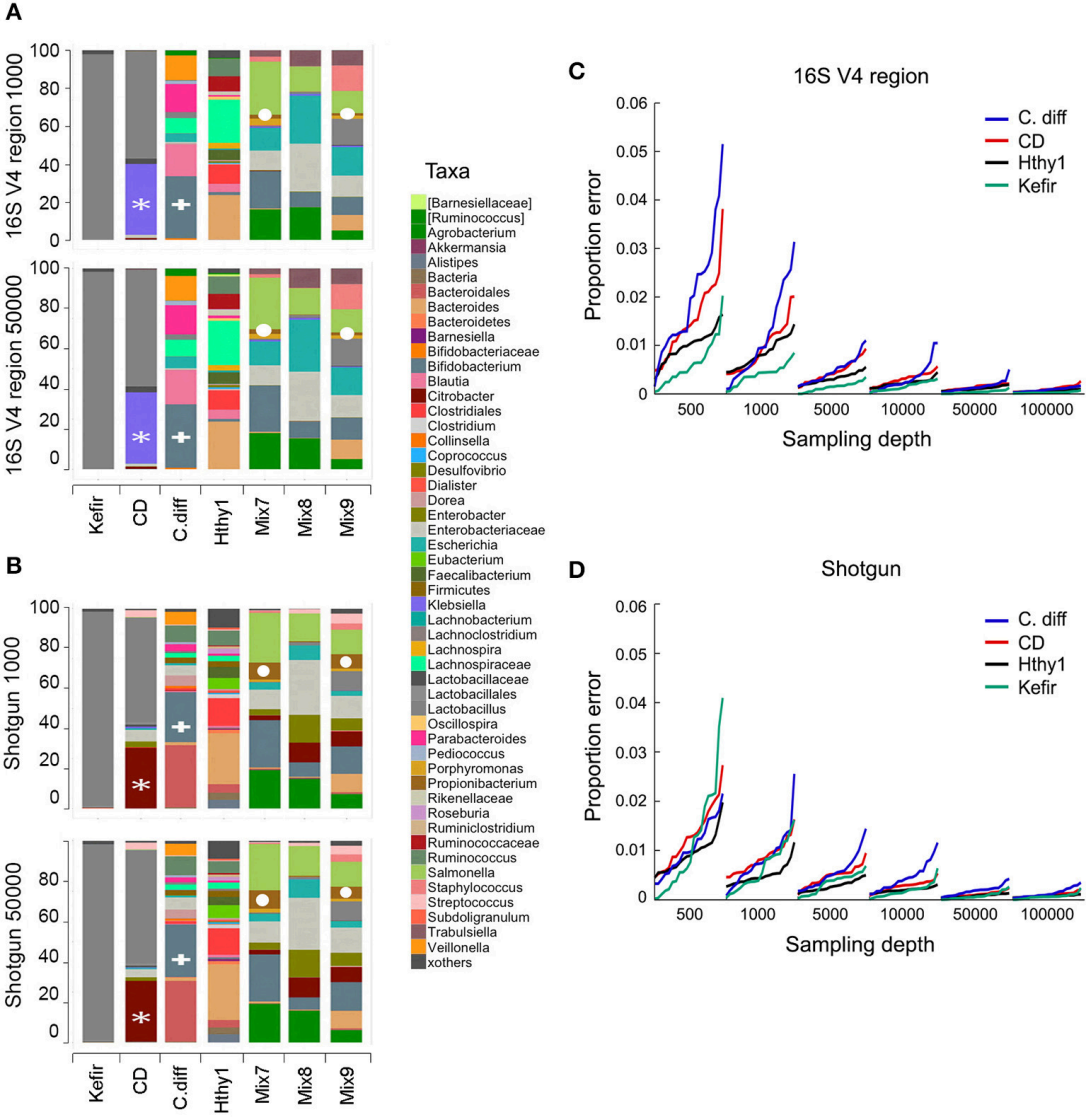
**Number of sequences required for taxonomic classification of samples with varying diversity**. A series of samples were chosen to assess the effect of library complexity on the accuracy of taxonomy assignments and estimation of diversity of bacterial populations. Kefir represents the lowest point in the bacterial diversity spectrum, followed by a patient affected by Crohn's disease (CD), another one recovered from *C. difficile* infection (C. diff), a healthy individual (Hthy1) and three artificial mixes of bacteria (Mix7-9). **(A,B)** Libraries were randomly sampled at depths of 500, 1000, 5000, 10,000, 50,000 and 100,000 reads. End1 16S rRNA gene sequences were classified with QIIME using the closed reference method to cluster OTUs and a similarity threshold of 97%. Paired-end shotgun metagenomics sequences were aligned with LAST and taxonomically classified with MEGAN5. Each random sampling was repeated 20 times. As an example, the relative abundance of taxa for one of these samplings at a depth of 1000 or 50,000 sequences is presented for 16S and shotgun metagenomics libraries. A white asterisk indicates a group of bacterial sequences identified as *Citrobacter* in the shotgun panel and *Klebsiella* in the 16S panel. *Bifidobacterium* is indicated with a white plus sign. *Propionibacterium* is indicated with a white circle. **(C,D)** For each taxa detected and for each random sample, the proportion error was calculated as the difference between the proportion that each taxon represented in the whole library (i.e., with the maximum number of reads) and in the random sample. This difference was weighted by the proportion that each taxon represented in the whole library. We present the arithmetic mean of all weighted differences for each of the 20 random samples.

To investigate the minimal sequencing depth sufficient for accurately profiling bacterial community composition, we randomly sampled our libraries at depths of 500, 1000, 5000, 10,000, 50,000, and 100,000 reads. At each depth, sampling and analyses were repeated 20 times. As an example, we show that the taxonomic classification for each type of library at sequencing depths of 1000 and 50,000 was surprisingly consistent (Figures 3A,B). It is expected that taxonomic classification performed with each method will be to some extent divergent, as the resolution of the sequences used for taxonomic assignments is distinct and variable depending on which region of the genome is captured in shotgun surveys, which variable region of the 16S rRNA gene is used, and which composition of species is present in the community under analysis. However, the general pattern of relative abundance of taxa was often observed to be similar although the concordance of 16s vs. shotgun methods was higher for simpler bacterial communities, as seen with the Kefir's community (Figures 3A,B). In the sample from the CD patient, the most abundant genus (*Lactobacillus*) was detected by both methods (gray bar), but the second was identified as *Klebsiella* in 16S and *Citrobacter* in the shotgun libraries (Figures 3A,B). This ambiguity likely occurs because the 16S rRNA gene sequences of these two genera share > 96% similarity. Many other taxa, like *Bifidobacterium* (Figures 3A,B) were consistently identified because they are phylogenetically more distant from the other taxa present. For the mock populations, all genera (*n* = 12) were found in shotgun libraries at both depths, but 16S libraries did not allow detection of the *Akkermansia* or *Clostridium* genera, even though they were ~5% of Mix-9. As expected, increasing sampling depth led to increased detection of taxa; with 1000 sequences 48 and 58 taxa were detected in 16S or shotgun libraries, respectively, and with 50,000 sequences this increased to 72 and 128. Based on our experimental bacterial mock populations, it is clear that some of the assignments are spurious and increasing sequencing depth augments the artifact. Of note, *Propionibacterium* was not included in our experimental mixes but was found in both types of libraries, indicative of contamination (Figures 3A,B). Indeed, environmental contamination poses a serious challenge for construction of NGS libraries (Laurence et al., 2014; Salter et al., 2014; Strong et al., 2014; Weiss et al., 2014).

Increasing the number of sequences results in more consistent estimations of bacteria relative abundance. To illustrate this point, we sampled reads from each library at various depths (500–100,000) and compared the proportion of each taxon to the full library for the Kefir, CD, C. diff., and healthy samples (Figures 3C,D). For each depth, we repeated sampling 20 times. We report the weighted arithmetic mean of the differences in proportion between the sampling and the full library. In general, the proportion error and its variance decrease with increasing sampling depth (Figures 3C,D). The number of sequences required per library will ultimately depend on the goals of the study and the type of analysis to be conducted (Ni et al., 2013).

In bacterial ecology, alpha (α) diversity refers to the species composition in sampling units, usually at a local scale (Whittaker, 1972; Lozupone and Knight, 2008). While the local scale concept is somewhat diffuse in population ecology, the compartmentalized nature of the human (or mouse) body creates well defined microbial communities (i.e., GI tract, mouth, etc.) on which α-diversity can be estimated for comparison purposes. We used the Shannon diversity and equitability indices (Shannon, 1948) as estimators of α-diversity for each of the random samples extracted from our libraries (Supplemental Figure 5). The Shannon diversity index is a sum of the proportion of each species relative to the total number of species in the community under analysis and therefore accounts for both abundance and evenness (Shannon, 1948). It was nearly identical at 1000 and 50000 reads with only a small variance over multiple repetitions for both 16S and shotgun libraries. The trend was the same for both methods: increasing Shannon diversity values were found from the Kefir sample, followed by the CD, *C. difficile*, and the sample from the healthy subject. As noted above, the Kefir microbiota only includes few species of bacteria and yeast (Supplemental Figure 4), and both CD and *C. difficile* infection have been reported associated with reduction of faecal bacterial diversity in the patients' stools (Chang et al., 2008; Antharam et al., 2013; Vincent et al., 2013; Kostic et al., 2014; Norman et al., 2015). This was well recapitulated by the Shannon diversity index (Supplemental Figure 5). The equitability index compares the actual diversity of a sample with the maximal possible diversity: the situation where all species are equally represented (Monte and Ghelardi, 1964). We found that the equitability decreased slightly with increasing sampling depth from 1000 to 50,000 reflecting the fact that previously unnoticed taxa were identified with increases in sampling depth.

## Comparing microbiomes by beta diversity

**Figure 4:**
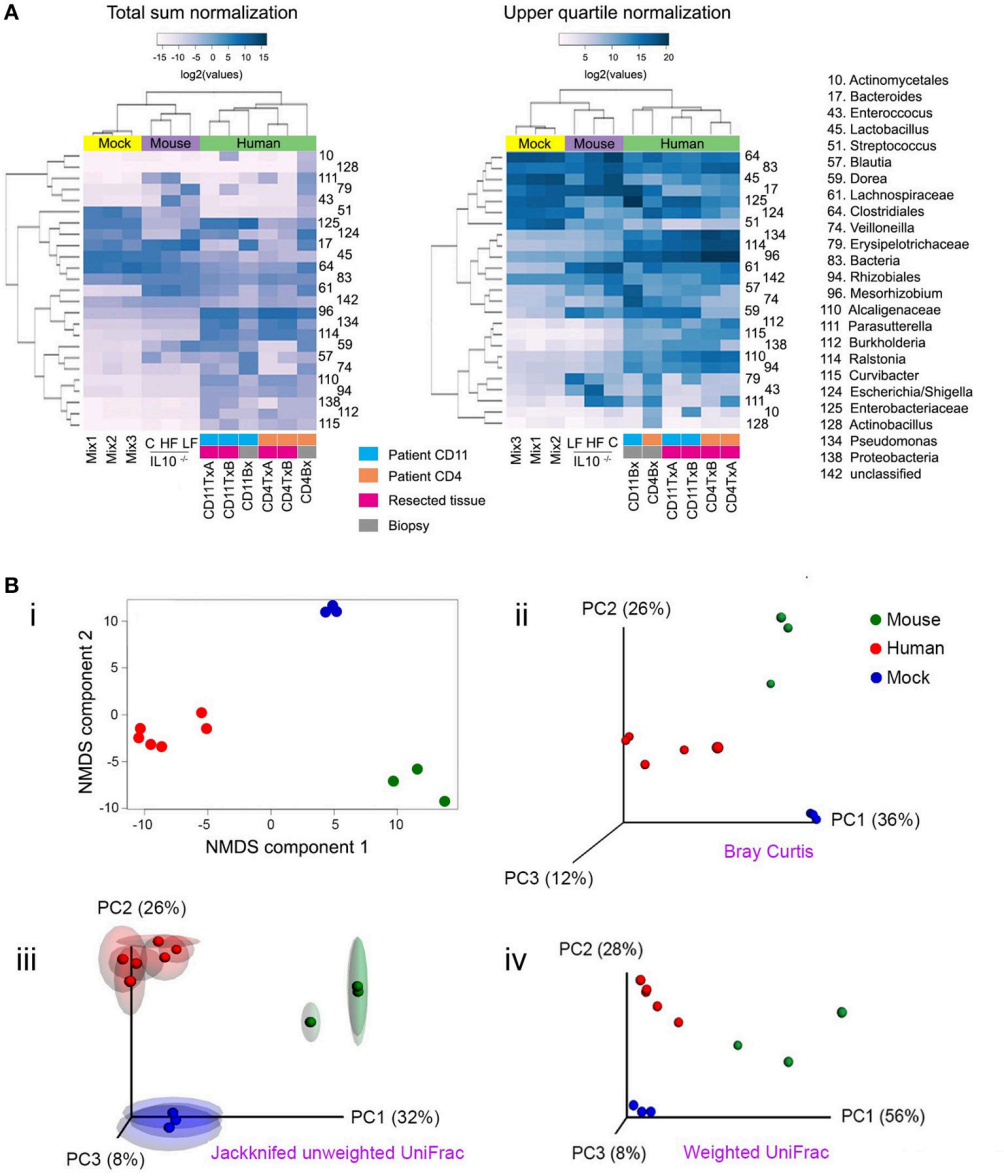
**Popular techniques for inspection and quantification of beta diversity. (A)** Heatmap of normalized counts for the 50 most abundant taxa. On top of the heatmap, group of samples are color-coded. Lilac (Mouse): mutant IL-10^−∕−^ mice that were fed with either high fat (HF), conventional chow (C) or low fat (LF) diet. Yellow (Mock): the three mock bacteria populations described in Figure 1. Light green (Human): samples from two patients suffering Crohn's disease (CD4 and CD11), including resections samples from the terminal ileum at the time of surgery (run in duplicate [**A,B**]) and biopsies taken 6 months after surgery. **(B)** Non-metrical multidimensional scaling (NMDS) and Principal Coordinates Analysis (PCoA). Upper panel: Bray-Curtis dissimilarities were ordinated and plotted by either NMDS **(i)** or PCoA **(ii)**. Lower panel: Unweighted **(iii)** or weighted **(iv)** UniFrac distances were analyzed and plotted by PCoA. For unweighted distances, jackknife resampling was performed and the spheres represent the average of such process while semitransparent ellipsoids represent the variance between repeats. Mix1-3 are described in the legend for Figure 1; IL10^−∕−^C: IL10 deficient mice fed with conventional chow diet; IL10^−∕−^HF: as previous one, but fed with high fat diet; IL10^−∕−^LF: as previous one but fed with low fat diet; CD11TxA: Patient 11 affected with Crohn's disease, tissue sample from ileocolic resection, repeat **(A)**; CD11TxB: as previous one, repeat **(B)**. CD11Bx: Biopsy from patient 11 colon, 6 months after resection. CD4TxA: Patient 4 affected with Crohn's disease, tissue sample from ileocolic resection, repeat **(A)**; CD4TxB: as previous one, repeat **(B)**. CD4Bx: Biopsy from patient 4 colon, 6 months after resection.

Beta (β) diversity considers the difference in bacterial community composition for different environments (Whittaker, 1972; Tuomisto, 2010). To illustrate some ideas and techniques related to beta diversity, we sequenced a set of 16S libraries that constitute three well-defined clusters of samples: three stool samples from mice fed with Chow, high fat or low fat diet; the three mock libraries described in Figure 1; and six ileum samples from two patients affected by Crohn's disease (CD). Users should be aware that clustering of samples that are highly disimilar would be more challenging than the illustrative set of data presented here, and will likely form less well-defined clusters. The analyses shown here are equally applicable to shotgun metagenomics data. Before any comparison can be made, the read counts (reads mapped to each taxon) must be normalized (Dillies et al., 2013; Paulson et al., 2013). In Figure 4A, we illustrate two popular normalization procedures: the total sum and upper quartile normalization. Respectively, for each sample, the normalization factor is the sum of counts of all bacterial taxa detected or the upper quartile value for each sample. In general, normalization procedures attempt to minimize the technical variability between samples, but also accounts for sample-specific dispersion (Dillies et al., 2013). Despite numerous research endeavors in this area, normalization remains a topic under intense debate, without a consensus on which normalization procedure is the most robust one (Paulson et al., 2013).

One commonly used method to detect discrete patterns of bacterial abundance in a group of samples is hierarchical clustering (Rokach and Maimon, 2005). Samples with similar bacterial profiles are recursively grouped together in branches of a dendrogram. Figure 4A presents the results of a hierarchical clustering using the complete linkage method (Rokach and Maimon, 2005). As expected, mice, experimental bacterial populations (mock), and human samples formed three discrete clusters (communities). Within the human samples, using total sum normalization, samples were clustered according to patient, and inside each patient ileal resections were separated from biopsies taken 6 months after surgery, when both patients presented with recurrent disease. With upper quartile normalization, biopsies were separated from resected tissues. Hierarchical clustering is a useful tool for visualizing co-abundance patterns, but in the absence of additional statistical tests, caution should be exercised as visual patterns can be misleading (Caporaso et al., 2010).

There are two main approaches for quantifying β-diversity: those that take into account the evolutionary differences between communities, formally known as phylogenetic β-diversity (Lozupone and Knight, 2005, 2008; Leprieur et al., 2012; Lozupone et al., 2013; Wang et al., 2013), and those that do not, formally known as taxon-based or non-phylogenetic methods (Kuczynski et al., 2010). With phylogenetic methods, differences in abundances that involve closely related species are given lower weights, on the assumption that closely related species have similar genetic capabilities. One example is UniFrac (unique fraction), which has been reported to correlate well with the biological properties of samples (Navas-Molina et al., 2013) and measures the amount of “unique evolution” of a community in comparison to others (Lozupone and Knight, 2005; Lozupone et al., 2006). Phylogenetic metrics are reliant on the quality of the constructed tree for the bacterial communities within the samples, which can be problematic in some cases, contingent on the taxa and the 16S rRNA gene variable region used. One of the most popular non-phylogenetic approaches to quantify β-diversity is the Bray-Curtis dissimilarity (Bray and Curtis, 1957; Beals, 1984). It is robust to the presence of zeroes in a count table, as often is the case for microbiome data (i.e., some bacterial taxa will be present in some but not all samples). QIIME and mothur offer the possibility to readily calculate many β-diversity metrics (Schloss et al., 2009; Navas-Molina et al., 2013) and so does the R package *vegan* (Oksanen et al., 2015).

Once distances/dissimilarities between samples (i.e., differences in bacteria abundance) have been computed, they can be positioned (ordinated) in a low-dimensional space (two or three orthogonal axes) to better appreciate how closely related they are to each other. The main assumption in all ordination methods is that there are a limited number of factors that greatly influence distribution and relative abundance of species. The two most commonly used ordination techniques in bacterial ecology are non-metric multidimensional scaling (NMDS) and principal coordinate analyses (PCoA), also known as metric multidimensional scaling (Quinn and Keough, 2002; Navas-Molina et al., 2013). The position of samples in the NMDS ordination represents the rank order of inter-sample distances, while in PCoA the ordination attempts to faithfully match their original inter-sample distances, providing results that are more readily interpretable (Ramette, 2007). In most cases, both techniques will lead to similar conclusions and it is a matter of debate which method is more appropriate (Ramette, 2007; Zur et al., 2007). For a more detailed discussion on multidimensional scaling see (Ramette, 2007; Zur et al., 2007; Buttigieg and Ramette, 2014). In Figure 4B, we illustrate both NMDS and PCoA analyses. In the upper panels, Bray-Curtis dissimilarities were calculated and are presented by (i) NMDS or (ii) PCoA. In the lower panels, we present UniFrac distances and PCoA ordination, either (iii) unweighted or (iv) weighted. Unweighted UniFrac considers presence/absence of OTUs and therefore emphasizes rare species, while weighted also considers the abundance of OTUs. The selection of each metric will depend on the hypothesis being evaluated as some phenotypes are more strongly influenced by relative abundance of taxa rather than presence or absence of specific taxa (Navas-Molina et al., 2013). As shown in Figure 4B(iii), it is possible to evaluate the stability of the PCoA plot using a resampling procedure known as jackknifing. For this procedure, calculations are reiterated after omitting one observation (taxa, OTU, etc.) and then the average is represented in a PCoA plot while the variance is depicted as confidence ellipsoids (Efron and Stein, 1981; Navas-Molina et al., 2013).

## Profiling the metabolic capacity of the microbiome

Determining the functional attributes of the microbiome is essential for understanding their role on host metabolism and disease (Joice et al., 2014). The metabolic capacity of the microbiome can be inferred or cataloged from 16S and shotgun metagenomics libraries, respectively. Gene marker approaches like 16S rely on the correlation between phylogenetic trees and clusters of genes shared between taxa (Langille et al., 2013). Shotgun metagenomics, on the other hand, provides a direct assessment of the functional attributes of the microbiome (Riesenfeld et al., 2004; Knight et al., 2012), although the results are dependent on sequencing depth.

The software PICRUSt (Langille et al., 2013) can be used to infer metabolic capacity of the microbiome contained in 16S libraries. PICRUSt functional inference is implemented in two steps. First, a reference phylogenetic tree is constructed from the Greengenes database (DeSantis et al., 2006) and gene contents are assigned to nodes in such tree if sequenced genomes are available, or otherwise predicted using ancestral state reconstruction algorithms (Langille et al., 2013). Representative sequences from OTUs derived from experimental data and associated with Greengenes identifiers are normalized by 16S rRNA gene copy number and then mapped to the corresponding Greengenes identifiers in the reference tree. The final result is an annotated table of gene counts per sample that can be linked to the Kyoto encyclopedia of genes and genomes (KEGG) orthology (KO) accession numbers (Kanehisa et al., 2004) or to any other orthologous protein family catalog. Similarly, several robust approaches have been developed to determine the functional attributes in shotgun metagenomics data, including MG-RAST (Meyer et al., 2008), MEGAN (Mitra et al., 2011), IMG/M (Markowitz et al., 2008), HUMAnN (Abubucker et al., 2012), and the R package ShotgunFunctionalizeR (Kristiansson et al., 2009). Using software like MEGAN5, each sequence can be directly mapped to KO representative sequences and the sum of KO counts that belongs to the same pathway can be computed. Alternatively, the SEED hierarchy (Overbeek et al., 2005) can be used to map reads to functional roles which can be organized into subsystems (Mitra et al., 2011). Thus, when normalized, results from PICRUSt and MEGAN5 are comparable. Recently, a new approach dubbed ShortBRED (Kaminski et al., 2015) was developed, which is both highly accurate and computer efficient. Essentially, it compiles a *de novo* database of marker peptides derived from reference databases and sequenced data, and then quantifies peptides abundance against such newly generated database.

**Figure 5:**
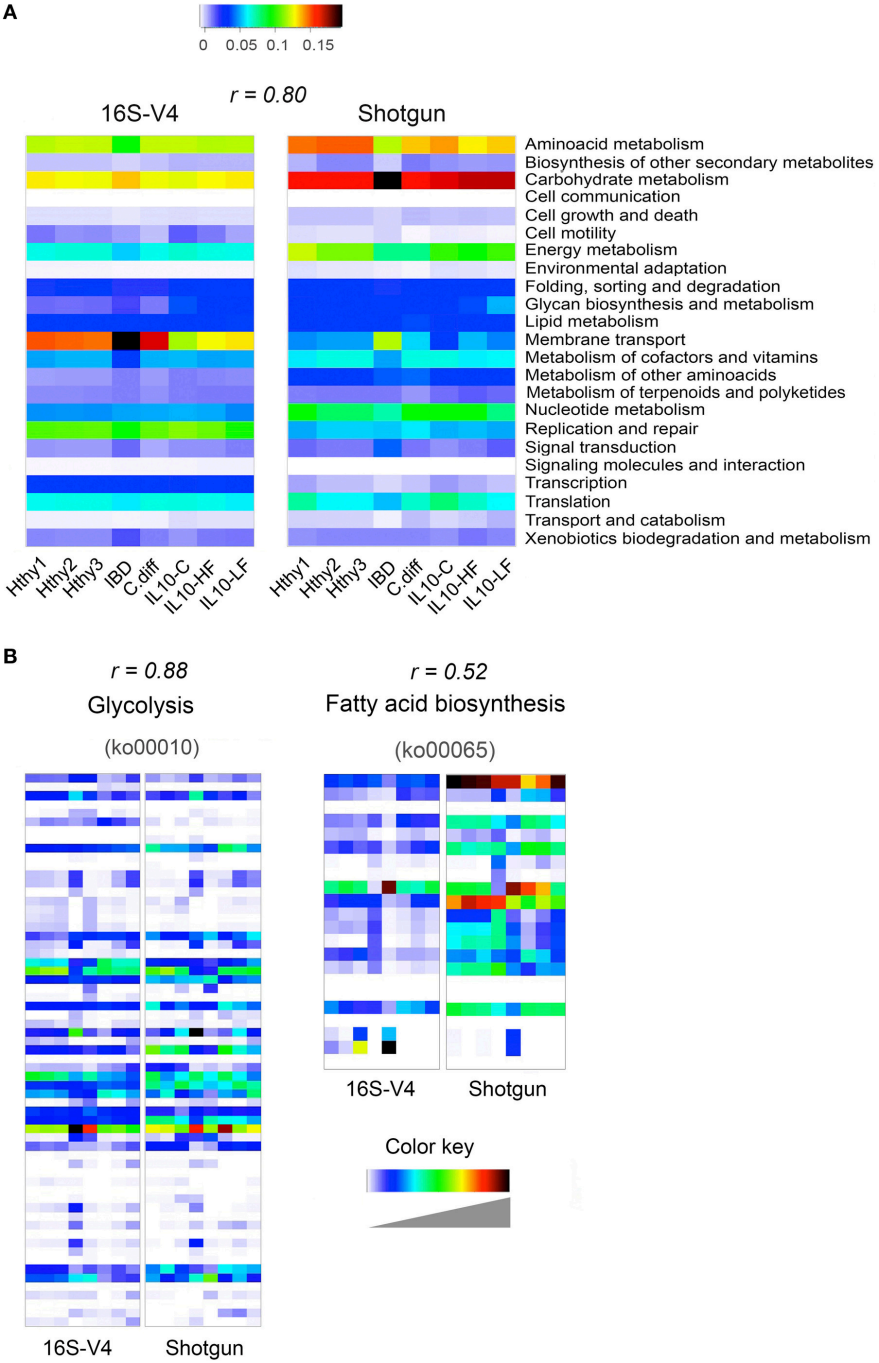
**Inference of gut bacterial microbiome functional content from 16S or shotgun metagenomics libraries**. Samples from three healthy individuals (Hthy1-3), the CD and the *C. diff* samples described in Figure 3, and the three mice samples described in Figure 4 were used here to illustrate metabolic inference of the gut bacteria microbiome from 16S or shotgun metagenomic libraries. High quality sequences were procured as described in Figure 1. **(A)** Twenty-three KEGG reference pathways known to be present in bacteria are depicted for both types of libraries. **(B)** Two KEGG pathways are illustrated at the gene (KEGG orthology, KO, groups) level. On top of each heatmap pair, the Pearson correlation coefficient for relative abundance of KOs derived with each method is presented. Inference of the functional content of the 16S metagenome was performed with PICRUSt, while gene content of shotgun metagenomic libraries was determined with MEGAN5. PICRUSt outputs results in number of bacteria cells that encode a gene (KO) while MEGAN5 outputs counts of sequences that mapped to a KO representative sequence. To make results from both methods comparable, counts were normalized by total sum. In both cases, the results represent the abundance of each KO as a fraction of the abundance of all detected KOs in each library. In order to achieve full representation of all values included in each normalized count table, colors in each heatmap were stretched between the minimum and maximum values. Therefore, the intensity (value) of each cell is not comparable between methods (16S of shotgun). Instead the Pearson correlation coefficient is shown as an estimator of the concordance of results provided by both approaches.

We derived functional profiles from 16S or shotgun libraries with PICRUSt or MEGAN5, respectively. For this analysis, we used stool samples from three healthy individuals, the CD and the *C. difficile* samples described in Figure 3, and the three mice samples described in Figure 4. Twenty-three KEGG reference pathways were used to compare relative abundance determined from both type of libraries (Figure 5A). The level of concordance between results derived from 16S or from shotgun metagenomics was variable depending on the pathway under consideration. In general both methods recapitulated general patterns of abundance. For example, the metabolic profile of the CD stool sample was clearly distinct from the rest and exhibited the highest gene content related to membrane transport, signal transduction and carbohydrate metabolism and the lowest content related to amino acid metabolism, metabolism of cofactors and vitamins and translation factors, as previously reported for IBD patients (Greenblum et al., 2012; Knights et al., 2013; Kostic et al., 2014). In addition, we show two KEGG reference pathways (at the KO level), which relative abundance was similarly (glycolysis; *r* = 0.88) or distinctly (fatty acid biosynthesis; *r* = 0.52) assessed by both programs (Figure 5B). The Pearson correlation coefficient of abundance of KOs detected by at least one of the methods was 0.66.

Although 16S and shotgun metagenomics both allow functional profiling of the microbiome, shotgun metagenomics offers a more reliable assessment, provided that enough sequences are available and, ideally, it should be complemented with metatranscriptomics analyses (Franzosa et al., 2015).

## Concluding remarks and perspective

The choice of shotgun or 16S approaches for microbiome analyses is usually dictated by the nature of the studies being conducted. For instance, 16S is well suited for analysis of large number of samples, i.e., multiple patients, longitudinal studies, etc. but offers limited taxonomical and functional resolution. Moreover, it should be pointed out that using primers for different regions of the 16S rRNA gene may lead to discordant results due not only to the distinct binding affinities for the corresponding flanking conserved regions, but also due to the resolution of each variable region across taxa (Soergel et al., 2012). Shotgun metagenomics on the other hand is usually more expensive but offers increased resolution, enabling a more specific taxonomic and functional classification of sequences as well as the discovery of new bacterial genes and genomes (Franzosa et al., 2015), which usually requires assembly of individual reads into contigs. Importantly, shotgun metagenomics allows the simultaneous study of archaea, viruses, virophages, and eukaryotes (Norman et al., 2014, 2015). Although several significant efforts to unravel bacterial strains have already been published (Qin et al., 2010; Qichao et al., 2014; Zhu et al., 2015), bacterial strains identification is an issue that remains unsatisfactory with current approaches. This is not only important from an aetiological perspective but also for the study of bacterial populations dynamics in general (Franzosa et al., 2015). Shotgun metagenomics offers a greater potential for identification of strains. Reportedly, the software MetaPhlAn2 has the ability to resolve different strains from the same species when reference genomes are available (https://bitbucket.org/biobakery/metaphlan2), and other software for shotgun data will likely perform well as more comprehensive databases are generated. Shotgun single-cell sequencing efforts also hold promise for bacterial strains deconvolution (Rinke et al., 2013).

In the view of experts in the field, metagenomics should be complemented with metatranscriptomics, proteomics, metabolomics and metadata, like clinical and dietary information, to derive mechanistic models that explain the structure and function of the microbiome (Brown et al., 2013; Morgan and Huttenhower, 2014; Franzosa et al., 2015; Waldor et al., 2015). Data integration will require sophisticated statistical techniques like ordination methods, hierarchical regression analyses, network analysis, and machine-learning approaches, among others (Abubucker et al., 2012; Segata et al., 2013; Franzosa et al., 2014; Joice et al., 2014; Morgan and Huttenhower, 2014). It is hoped that this primer will provide clinicians and researchers with a basic understanding of the main bioinformatics approaches for microbiome analyses with a view of advancing future investigations.

## Author contributions

JJ, JP, NH, AM, KM, GW designed the study. NH, SO, TM performed experiments. JJ, JP, WW performed bioinformatics analyses. TP, DK contributed clinical samples. JJ, AM, KM, and GW wrote the manuscript. All authors read and approved the manuscript.

## Funding

This work was supported by funding from Alberta Innovates Technology Futures-Innovates Centers of Research Excellence (AITF-iCORE) to GW and Canadian Institutes for Health Research and Alberta Health Services to KM.

### Conflict of interest statement

The authors declare that the research was conducted in the absence of any commercial or financial relationships that could be construed as a potential conflict of interest.

## Supplementary material

Supplementary Figure 1 **Average quality scores of mock libraries presented in Figure 1 show that end1 (blue line) is higher than the corresponding quality in end2 (red line) especially at the 3′ end of each end**.

Supplementary Figure 2 **Comparison of taxonomic classifications using different combinations of end1 and end2 for 16S libraries**. The name of the method is at the bottom of each bar and the Pearson correlation coefficient between the expected (Input) and obtained relative abundance of taxa is on top of each bar.

Supplementary Figure 3 **Taxonomical classification of reads from library Mock1 described in Figure 1**. Sequences were aligned either with BLAST (upper panel) or LAST (lower panel) and taxonomically classified using MEGAN5. The Krona plot depicts different bacteria taxonomic levels in concentric circles, from subspecies in the outermost circle to the bacteria kingdom in the innermost circle.

Supplementary Figure 4 **Taxonomical classification of sequences derived from the Kefir shotgun metagenomics library described in Figure 3**. Taxonomical classification was done by alignment of sequences to the NCBI nt/nr and human microbiome project bacteria databases and then classified using MEGAN5.

Supplementary Figure 5 **Shannon's diversity index was used to describe species diversity in each bacterial community (the so-called α-diversity)**. It takes into account the number of species and their evenness, and is calculated as a weighted sum of the proportion (*p*) that each species (*i*) constitutes of the total number of species (*S*) in the bacterial community ($H=-\sum_{i=1}^{S}pilnpi$). The higher the number of species and number of individuals inside each species, the higher the Shannon's diversity index will be. Shannon's equitability or evenness (E_*H*_) index compares the actual diversity with the maximal possible diversity (the situation when all species are equally abundant), and is calculated as (*E*_*H*_ = *H*∕*H*_*max*_ = *H*∕*lnS*). A bacterial community in which all species are equally represented will have an equitability of 1. The average value of both indices and the corresponding standard deviation were calculated from 20 simulations at depths of 1000 (blue boxes) and 50,000 (magenta boxes) reads. Artificial bacterial mixes were excluded. Kefir, grains of Kefir; CD, sample from a patient affected by Crohn's disease; Cdiff, sample from a patient affected by *Clostridium difficile*; Hthy1, sample from a healthy individual.

## Glossary

α-diversity: number of species (richness) at one site. Some formulations also consider the proportion that each species represents (evenness), in which case a high diversity implies a large number of species with similar abundances.
β-diversity: differences in species composition between sites. Some formulations incorporate phylogenetic information, assigning lower weights to differences in abundances that involve closely related species, on the assumption that closely related species have similar genetic capabilities.
Bray Curtis dissimilarity: a non-phylogenetic measurement of β-diversity based only on the species present in both sites.
Greengenes: a database of 16S rRNA gene sequences at the Lawrence Berkeley National Laboratory.
Hierarchical clustering: a method to detect patterns of bacterial abundance by recursively grouping samples with similar bacterial profiles into branches of a dendrogram.
Lowest common ancestor (LCA): refers to the common node of two descendants in a phylogenetic tree. With respect to taxonomic classifications, assignments are made at the lowest non-ambiguous level.
Non-metric multidimensional scaling (NMDS): an ordination method where the positions on the low-dimensional plot represent the rank orders of the inter-sample distances.
Operational taxonomy unit (OTU): a group of sequences clustered together based purely on similarity and an arbitrary threshold. OTUs may or may not be equivalent to taxonomical entities (species, genera, etc.).
Ordination: statistical techniques to transform multi-dimensional datasets into easier-to-visualize two or three-dimensional representations, such that similar datasets are placed close to each other and dissimilar datasets are placed far from each other.
Principal coordinate analyses (PCoA): an ordination method where the relative positions on the low-dimensional plot attempt to faithfully match the original inter-sample distances.
Read counts normalization: a linear scale factor correction that facilitates dataset comparisons by minimizing technical sources of variability and sample-specific data dispersion patterns.
UniFrac: a measurement of β-diversity that incorporates the phylogenetic distances between species. Both weighted (quantitative) and unweighted (qualitative) variants are used, where the former accounts for abundance, while the latter only considers presence vs absence.
Unique clad-specific marker genes: genes that are universally found in their taxonomic clade and yet are absent outside it, as scored by BLAST. Typically about 5% of bacterial genes will qualify
