# Supplementary figures and images for: Characterization of the Gut Microbiome Using 16S or Shotgun Metagenomics
Source: Front Microbiol. 2016 Apr 20;7:459. doi: 10.3389/fmicb.2016.00459 (PMC4837688; doi:10.3389/fmicb.2016.00459)

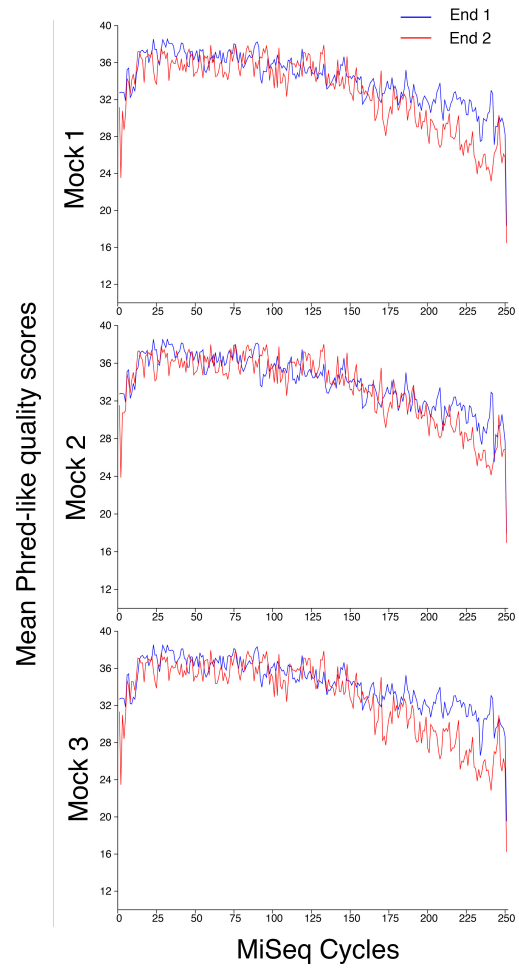

Supplmental Figure 1

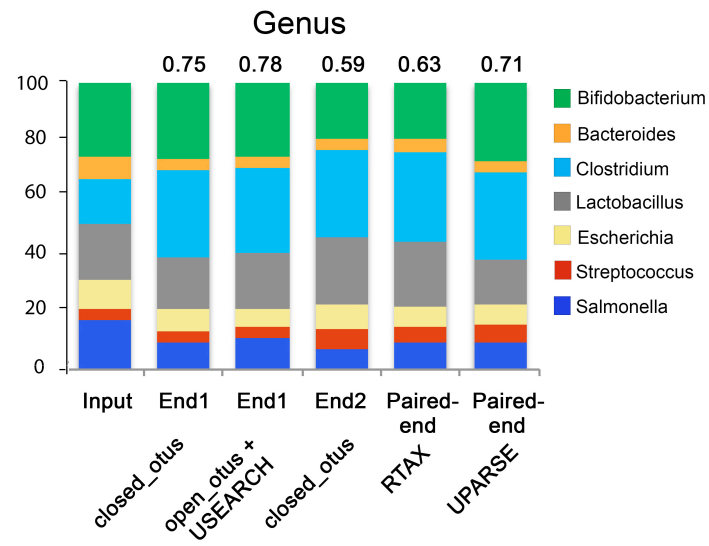

Supplemental Figure 2



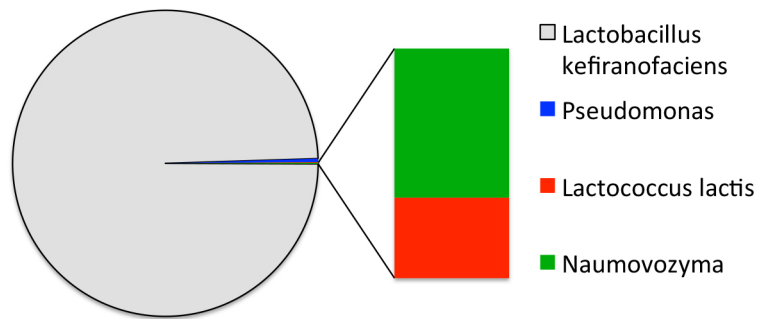

Supplemental Figure 4

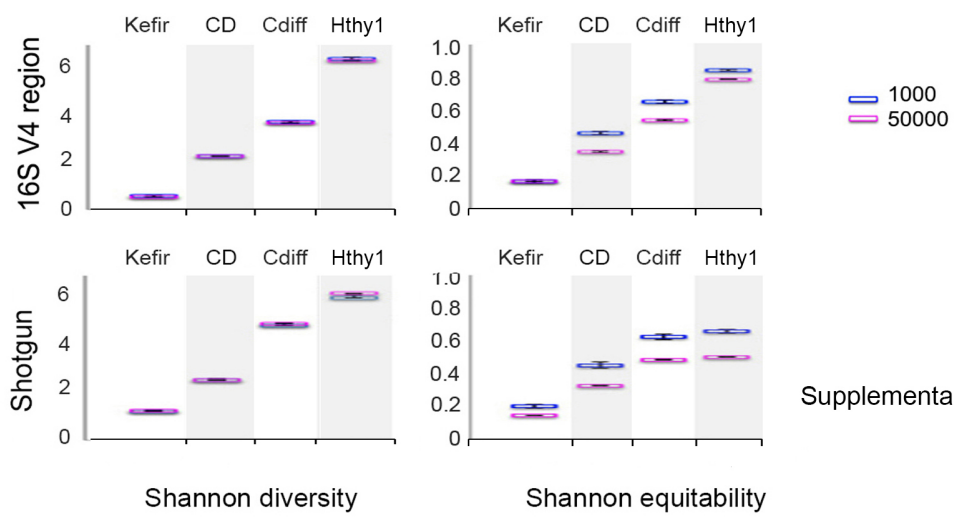

Supplemental Figure 5

Supplement: Supplementary Figure 1 — Average quality scores of mock libraries presented in Figure 1 show that end1 (blue line) is higher than the corresponding quality in end2 (red line) especially at the 3′ end of each end. [file Image1.PDF]
